# Benchmark dataset for undirected and Mixed Capacitated Arc Routing Problems under Time restrictions with Intermediate Facilities

**DOI:** 10.1016/j.dib.2016.06.067

**Published:** 2016-07-06

**Authors:** Elias J. Willemse, Johan W. Joubert

**Affiliations:** Center of Transport Development, Department of Industrial and Systems Engineering, University of Pretoria, 0002, South Africa

**Keywords:** Optimisation, Waste management, Operations Research, Capacitated Arc Routing Problems

## Abstract

In this article we present benchmark datasets for the Mixed Capacitated Arc Routing Problem under Time restrictions with Intermediate Facilities (MCARPTIF). The problem is a generalisation of the Capacitated Arc Routing Problem (CARP), and closely represents waste collection routing. Four different test sets are presented, each consisting of multiple instance files, and which can be used to benchmark different solution approaches for the MCARPTIF. An in-depth description of the datasets can be found in “Constructive heuristics for the Mixed Capacity Arc Routing Problem under Time Restrictions with Intermediate Facilities” (Willemseand Joubert, 2016) [2] and “Splitting procedures for the Mixed Capacitated Arc Routing Problem under Time restrictions with Intermediate Facilities” (Willemseand Joubert, in press) [4]. The datasets are publicly available from “Library of benchmark test sets for variants of the Capacitated Arc Routing Problem under Time restrictions with Intermediate Facilities” (Willemse and Joubert, 2016) [3].

**Specifications Table**

**Table:** 

Subject area	*Optimisation, Waste Management*
More specific subject area	*Operations Research, Capacitated Arc Routing Problems*
Type of data	*Table, figures, text files*
How data was acquired	*A Geospatial Information System (GIS) data set, courtesy of Business Connexion, was used to generate the Cen-Full-IF, Cen-IF and Cen-Part instances. The Act-IF instances were generated using OpenStreetMap data and information supplied by the Metropolitan municipality responsible for servicing the area. All other instances were generated by modifying publicly available datasets.*
Data format	*Raw*
Experimental factors	*The Cen-IF instance files were generated by transforming a GIS dataset data into a connected road-segment network, and calculating waste and traversal data for the network using road segment attributes. Thereafter the network data was converted into a standard instance file format. An illustration of the file preparation can be found in Fig. 1. The Lpr-IF instances were generated by modifying existing raw instance files, and transforming them into the standard instance file format.*
Experimental features	*The datasets are used to evaluate solution techniques for the CARPTIF and MCARPTIF.*
Data source location	*Centurion (25.860°S 28.189°E), South Africa. Actonville (26.214°S 28.304°E) and Wattville (26.222°S 28.303°E), Benoni, South Africa.*
Data accessibility	*The datasets are available in this article and at*http://dx.doi.org/10.17632/9x4vd92rcj.2

**Value of the data**

• The benchmark sets include realistic instances that are based on actual road networks and that are consistent in size with waste collection instances found in practice. They can therefore be used to evaluate the performance of solution methods intended for practical applications.
• The instances can be used to benchmark and compare existing and new solution approaches for the CARPTIF and MCARPTIF, including lower-bound procedures, as well as exact and heuristic solution methods.
• Characteristics of the realistic benchmark sets can be compared against other practical waste collection instances to identify common instance characteristics that may influence the performance of solution methods.
• The large instances can be used to evaluate solution methods for tactical and strategic waste collection problems, such as waste collection sectoring, intermediate facility placements, and vehicle fleet composition problems.
• The instances can be reduced to and solved as the Capacitated Arc Routing Problem (CARP) and Mixed Capacitated Arc Routing Problem (MCARP), thus extending their use to other variants.

## 1 Data

The data accompanying this paper consist of benchmark test instances for the Capacitated Arc Routing Problem under Time duration restrictions with Intermediate Facilities (CARPTIF), as well as files for the Mixed CARPTIF (MCARPTIF) on a mixed road-network with one- and two-way streets. The problems are generalisations of the Capacitated Arc Routing Problem (CARP), and closely represents waste collection routing. All datasets are freely available from [3].

## 2 Experimental design, materials and methods

**Fig. 1:**
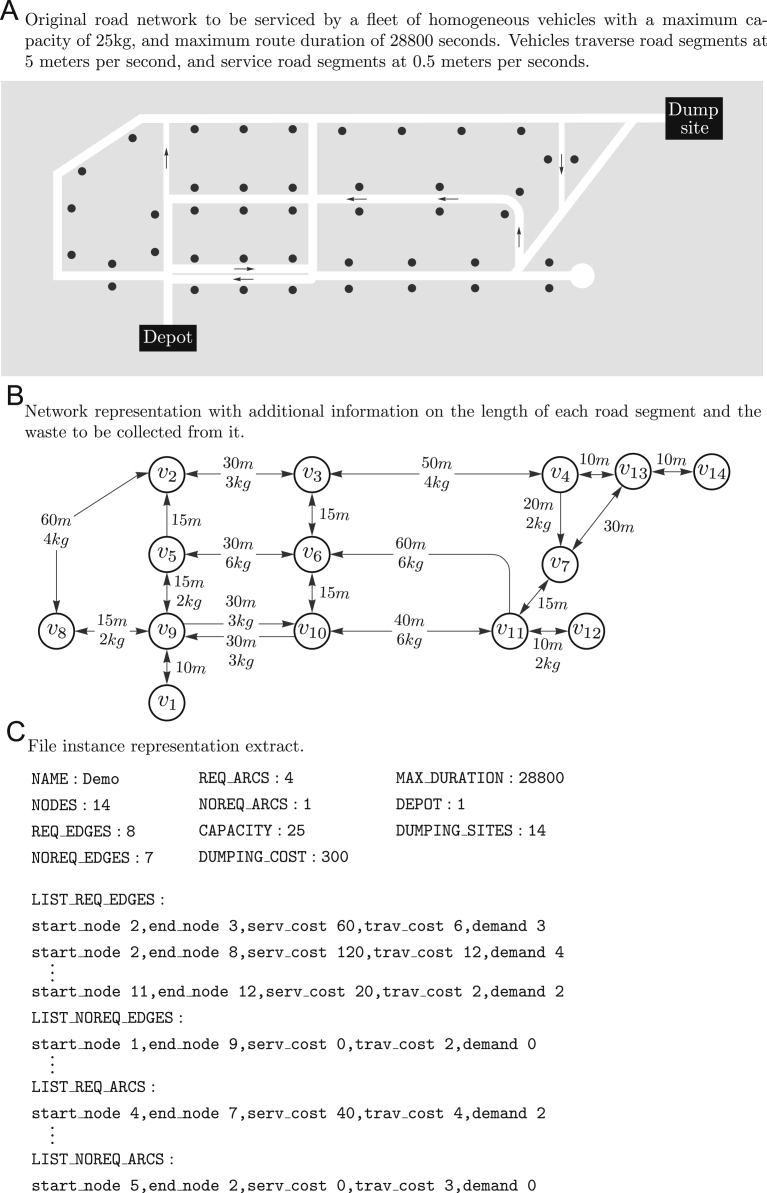
Transformation from real road network data to a benchmark instance file.

In this section we describe in the process followed to generate the different datasets. The generic process of converting a waste collection instance into a benchmark file is illustrated in Fig. 1. A detailed description of the instance format is available in [3].

### 2.1 Cen-IF-Full, Cen-IF and Cen-Part-IF instances

The *Centurion (Cen)* MCARPTIF sets were developed in this paper and is used for computational tests in [2], [4].

To generate the *Cen-IF-Full, Cen-IF* and *Cen-Part-IF* instances, a Geospatial Information System (GIS) data set of the Centurion area, courtesy of *Business Connexion*, was used to create the benchmark instances. The data set accurately describes the network and includes a number of useful attributes. Accurate deadheading costs, service costs and waste quantities are not available for the network, so we inferred the arc-routing data using a similar approach to that of [1]. Though the metadata for the Centurion files are fabricated, the actual road network data are not. The municipality of Centurion has to service the entire road network, so the large files are representative of actual waste collection instances.

In the original GIS data set all road segment centerlines are represented by polylines, which, in turn, is made up out of no less than two nodes (or points). We inferred the origin node, denoted by *FromNode*, as the first node in the polyline description, and the destination node, denoted by *ToNode*, as the last node. We refer to the *(FromNode, ToNode)* combination as a link.

Associated with each link is a *ONEWAY* field that has one of three values: ‘*B׳* indicates that it is a bi-directional road segment; *‘FT’* indicates a one-way in the direction from the *FromNode* to the *ToNode*; and *‘TF’* indicates a one-way in the direction from the *ToNode* to the *FromNode*. We inferred the link to be an arc if it has a field value of either *‘TF’* or *‘FT’*, and an edge if the field value is ‘B’.

A road category field identifies the road type within the network hierarchy. If the link has a field value of type ‘STREET’ or ‘OTHER׳, we assigned a value 1 to the link, or a value 2 if the type is *‘DUAL CARRIAGEWAY’*, ‘*NATIONAL HIGHWAY’*, *‘NATIONAL ROAD’*, *‘MAIN ROAD’* or *‘RESTRICTED ACCESS ROAD׳*. Both sides of a type 1 link were assumed to be serviceable in a single traversal. If a link is either an arc or edge with a type 1 value, it will remain a single link. We assumed that the two sides of a type 2 links, on the other hand, must be serviced separately since it would be either dangerous, or physically impossible to have refuse collected on both sides of the road. An example would be a busy suburban road with two lanes in either direction. Links of type 2 that are arcs were then replaced with two arcs, both in the direction of the original arc, each arc representing one side of the road. Type 2 links that are edges were replaced with two directed arcs, one in each direction.

Links, both arcs and edges of either type 1 or 2, with a speed limit exceeding 60 km/h were assumed to have no demand. Links with a speed limits of at most 60 km/h were assumed to have demand. Demand of 10 kg per household, and one household each 20 m were assumed. In the case of type 1 links the demand was doubled since both sides of the road was assumed to have demand. Demand was then calculated using the following equation

$$demand=\frac{d}{20}\times m$$

where *d* denotes the length of the link, and *m* the multiplication factor of 2 if it is of type 1, and 1 if it is of type 2. Road segments with a category other than those listed were assumed to be of type 1, but with no demand. The traversal cost of a link was determined as the time it takes to traverse the link at a speed of 20 km/h. To derive the service cost, we added a loading time of 10 s per bin (10 kg or part thereof) to the traversal cost.

For the instances we imposed a maximum vehicle capacity of 10 t (10,000 kg) and a maximum route duration of 8 h (28,800 s). For each street segment in the network we modelled separate deadheading and collection times. We also assumed that an Intermediate Facility (IF) visit incurs a cost of 300 s. All cost values in the file instances are in seconds, and demand values in kilograms.

**Fig. 2:**
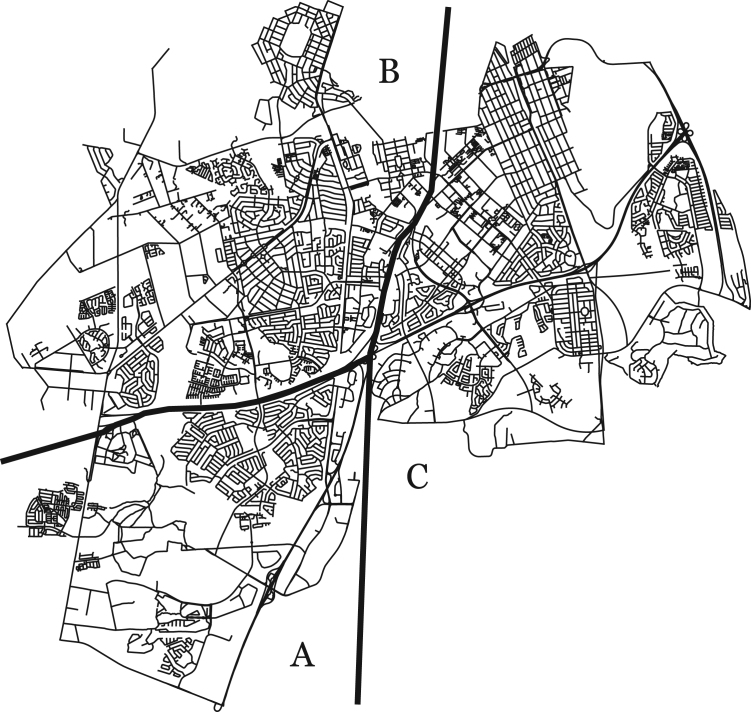
Centurion area road network used to generate the Cen-Full-IF, Cen-IF and Cen-Part-IF instances (source [8]).

Fig. 2, taken from [2], shows the road network of the *Cen-IF-Full* instance, as well as the *a*, *b* and *c* areas used to generate the three *Cen-IF-a, Cen-IF-b* and *Cen-IF-c* instances. The four *Cen-Part-IF* instances were generated by further subdividing the *Cen-IF-b* and *Cen-IF-c* instances into *Cen-IF-b-1, Cen-IF-b-2, Cen-IF-c-1,* and *Cen-IF-c-2* instances. The pair of subdivided instances contain the full network of the original instance, but with the required arcs and edges distributed between the two subdivided instances. The division was done on the raw instance file in such a way that the pair of subdivided instances have approximately the same number of required arcs and edges.

### 2.2 *Lpr-IF* instances

The *Lpr-IF* MCARPTIF set was developed in this paper by extending the existing *lpr* Mixed CARP (MCARP) sets of [1], available from http://www.uv.es/belengue/mcarp/, and is used for computational tests in [2], [4]. The original set consists of 15 instances with arc traversal and service costs and arc demands given in seconds and kilograms, respectively. The MCARP instances were transformed into MCARPTIF instances by including a route duration limit of 28,800 s, and by including IFs at nodes ⌊|V|/2⌋ and 2⌊|V|/2⌋, where |V| is the number of nodes in the instance file.

## References

[1] Belenguer J., Benavent E., Lacomme P., Prins C. (2006). Lower and upper bounds for the mixed capacitated arc routing problem. Comput. Oper. Res..

[2] Willemse E., Joubert W. (2016). Constructive heuristics for the Mixed Capacity Arc Routing Problem under Time Restrictions with Intermediate Facilities. Comput. Oper. Res..

[3] E. Willemse W. Joubert Library of benchmark test sets for variants of the Capacitated Arc Routing Problem under Time restrictions with Intermediate Facilities, v2, Mendeley Data, DOI: 10.17632/9x4vd92rcj.2. Available online from http://dx.doi.org/10.17632/9x4vd92rcj.2 10.1016/j.dib.2016.06.067 PMC4961348 27508252

[4] Willemse E., Joubert J. (2016). Splitting procedures for the Mixed Capacitated Arc Routing Problem under Time restrictions with Intermediate Facilities. Oper. Res. Lett..

